# Awareness of the Ayushman Bharat-Pradhan Mantri Jan Arogya Yojana in the Rural Community: A Cross-Sectional Study in Eastern India

**DOI:** 10.7759/cureus.35901

**Published:** 2023-03-08

**Authors:** S S V Prasad, Chandramani Singh, Bijaya N Naik, Sanjay Pandey, Rajath Rao

**Affiliations:** 1 Community and Family Medicine, All India Institute of Medical Sciences Patna, Patna, IND

**Keywords:** health insurance, utilization, impact, health care expenditure, coverage, ayushman bharat scheme, awareness

## Abstract

Background

Ayushman Bharat-Pradhan Mantri Jan Arogya Yojana (AB-PMJAY) is one of the flagship programs intended to provide financial protection to people availing of secondary and tertiary level health care. It’s been nearly three years since the implementation of AB-PMJAY in Bihar, yet the level of awareness, especially in rural communities, is unknown. So, this study was planned to explore the awareness and utilization of AB-PMJAY in the selected rural area of Bihar.

Methodology

This community-based cross-sectional study used a multistage sampling strategy to select 802 households within a radius of 5 Km from the rural health training centre, Naubatpur. A pre-tested, semi-structured questionnaire was used to collect the necessary data regarding awareness of AB-PMJAY. The association between category, occupation, education, age group, and ration card with the awareness of AB-PMJAY was assessed using Pearson’s chi-square test.

Results

The awareness of the AB-PMJAY was 68.6% (95% CI: 65.30%-71.7%), while out of 459 eligible study participants, 362 (78.9%) were aware of the AB-PMJAY. Utilization of AB-PMJAY was only 1.3% among the eligible study participants. There was a statistical significance between the category of eligible study, ration card, and employment status with the awareness of the AB-PMJAY.

Conclusion

Every two out of three rural individuals and three out of every four eligible participants were aware of the AB-PMJAY scheme, while the level of utilization was found to be very low at 1.3%; hence, training of the healthcare workers at the grass-root level, like accredited social health activists (ASHA) and Anganwadi workers (AWW), should be done regularly to improve the connection in the community and for effective utilization of the Ayushman Bharat-PMJAY.

## Introduction

In India, 3.8% of the gross domestic product (GDP) is spent on healthcare expenditure, and out-of-pocket expenditure accounts for 58.78%. As per the National sample survey office (NSSO) 75th round report, about 55% of the Indian population (rural: 52% and urban 61%) avail of healthcare services from the private sector. In rural areas, almost INR 15,937, and in urban areas, INR 22,031 are spent as out-of-pocket medical expenditures for hospitalization [1]. Health care expenses push many families into debt, as most of the Indian population belongs to the middle class or lower socio-economic class. India is a developing country with an expanding population of 1.4 billion [2], and Bihar is one of the most populous states/UTs in India [3]. Bihar is among India's worst-performing states in terms of providing health care services, as reflected by health indicators [4]. The proportion of families living below the poverty line in Bihar is 52.2% [5]. There are various models of health care, and the out-of-pocket model is the most prevalent in less-developed regions and countries where there are insufficient financial resources to construct a medical system similar to the other three models discussed previously. The wealthy receive expert medical treatment, while the poor do not unless they are able to come up with the funds to pay for it [6].

A United Nations high-level meeting suggested universal health coverage to prevent healthcare cost inequity with unequal access to health care [7]. The current universal health coverage (UHC) movement began in response to growing worldwide awareness of issues such as limited access to health care, substandard treatment, and significant financial risk [7]. To help decrease this spending and provide adequate health care, the Indian government, which includes both state and central governments, has implemented several health security programs, including Ayushman Bharat, a fully central government-funded scheme [1]. This initiative was inaugurated in Ranchi on September 23, 2018, by the honorable Prime Minister of India. The aim of implementing the vision of universal health coverage was to ensure that no one is left behind [1]. Ayushman Bharat is one of the flagship programs intended to provide financial protection in availing of secondary and tertiary level health care. This scheme has two components: the health and wellness center and the Pradhan Mantri Jan Arogya Yojana (PMJAY). PMJAY is the world’s largest public-funded health insurance scheme and applies to India’s rural and urban populations. PMJAY’s primary purpose was to reduce catastrophic out-of-pocket expenses, cover the most amount in the least amount of time and improve prolonged hospitalization [8].

Various health insurance schemes like the Rashtriya Swasthya Bheema Yojana (RSBY) [9], the Aam Aadmi Bheema Yojana [10], the Central Government Health Scheme [11], etc., are in full swing before the launch of the Pradhan Mantri Jan Arogya Yojana (PMJAY) health insurance scheme in India. However, many health insurance schemes have some or other limitations. For example, RSBY has a cap on family size and the cost of care (INR 30,000), and OPD cost has increased in the RSBY family by 23% [12].

The awareness level of PMJAY in the rural areas of Bihar was found to be only 6.7% [13]. While other individual studies in India also reported a low level of awareness [13,14]. There is a dearth of literature in the community-level assessment of the awareness of Ayushman Bharat-PMJAY in India. Very limited studies have assessed PMJAY at the community level [15,16]. As per data from the government of Bihar, in the year 2020, only 50% of the population was eligible for Ayushman Bharat [17]. It has been nearly three years since the implementation of Ayushman Bharat-PMJAY in Bihar, which signifies that there will be a change in the level of awareness. Hence, this study is intended to explore the utilization and awareness of Ayushman Bharat-PMJAY in a selected rural area of Bihar.

## Materials and methods

Study settings

The study was carried out in the rural area of Patna, the capital of the state of Bihar, along the south bank of the river Ganges in eastern India. Patna has a total of six medical colleges, excluding the other various healthcare centers. This study was conducted in villages within a 5 km radius of the rural field practice area of a tertiary health care facility, Patna, run by the Department of Community and Family Medicine in Naubatpur block, which has a population of 203,594 (Census 2011), residing in 110 villages [3]. The rural health training center (RHTC) of the tertiary health care facility works in coordination with the Bihar health department and provides services in the Naubatpur block villages. 

Study design

This study adopted the community-based cross-sectional study design for six months (July 2021-December 2021).

Study participants

The study population included heads of families who are permanent residents or have resided for a minimum period of one year in the villages within a 5 km radius of RHTC Naubatpur, and families not giving consent to participate were excluded from the study. 

Sample size and sampling technique

Since there was a lack of literature, the awareness of Ayushman Bharat was assumed to be 50% among beneficiaries, and the sample size was calculated to be 401 using OpenEpi software at a 95% confidence interval, a 6% absolute margin of error, and a design effect of 1.5. Ayushman Bharat beneficiary contributes to 50% of the total population in Bihar [17]. Since the study is done among the general population, the final sample size was calculated to be 802 (double the calculated size).

We adopted a multi-stage sampling technique. In the first stage, eight villages were conveniently selected from a total of 12 villages that were present within a radius of 5 km from the RHTC: four villages from the 0-2.9 km radius of the Naubatpur RHTC and four villages from the 3-5 km radius of the RHTC. In the second stage, the total number of households that should be taken from both groups' villages was stratified according to the number of total households in that village proportionately, i.e., in a total of 3680 (48.02%) households are there in a 0-2.9 km distance radius and 3982 (51.97%) households in a 3-5 km range, so we had to take 384 households from the villages up to 3 km, and 418 households were selected from villages in 3-5 km range, which comes to a total of 802. In the third stage, a total enumeration list of all households was done, and then the houses were selected using systemic random sampling, in which every fifth house was taken for the study as a sampling interval. After selecting the houses, the heads of the households were approached, and information was gathered about their socio-demographic characteristics and their awareness of the Ayushman Bharat health insurance plan. If the head of the household refuses to participate in the study or is not available in the house, or if the house is locked even after two visits for prior information, then the house next in line is taken for the study.

All 802 participants (100% response rate) took part in the study. 

Study tool

We used the predesigned, pretested, semi-structured study tool to collect the information from the study participants. The questionnaire was developed initially in English and converted into Hindi with the help of a medico-social worker and principal investigator. It was piloted among the 30 households in Moti Nagar village of the representative sample population, and the questionnaire was modified per the needs of the community with the assistance of subject experts. The questionnaire was back-translated to Hindi for administration. The process was according to the World Health Organization Disability Assessment Schedule Translation Package (WHODAS 2.0 TRANSLATION PACKAGE) protocols for language translation [18]. The Cronbach alpha was calculated for the same and came to be 0.8 (good reliability). The study tool was divided into various sections. Section A consisted of socio-demographic details of the participants like the name of the respondent, family ID, family table, total income of the family, per-capita income, socio-economic status using the Modified BG Prasad classification [19], and presence of a ration card [according to the Public Distribution System (PDS) of Bihar] [20]. The category of the family, catastrophic expenditures, and debts, if any, of the family. Section B comprised the items related to the awareness of the Ayushman Bharat, the level of awareness regarding the various types of health insurance given by the government, specifically regarding the Ayushman Bharat, and the specific use of the PMJAY, and awareness and utilization regarding the Ayushman Bharat health card.

Study procedure

After getting approval from the Institute Research Committee (IRC) and Institute Ethics Committee (IEC) of AIIMS Patna in July 2021, this study was conducted. The data collection process began as soon as the questionnaire was completed and approved. The data collection was done by the principal investigators along with the co-principal investigators with the help of medico-social workers. The consent from the head of the family was taken before they were interviewed face-to-face in the local language (Hindi) to determine their level of awareness of the Ayushman Bharat health insurance program. During the interview, information was gathered about their socio-demographic characteristics and their awareness and utilization of the Ayushman Bharat health insurance plan. Throughout the study, privacy and confidentiality were maintained. The collected information was checked for correctness and completeness every fortnightly.

Statistical analysis

The data was cleaned, and important variables were classified. IBM Corp. Released 2013. IBM SPSS Statistics for Windows, Version 22.0. Armonk, NY: IBM Corp. was used to statistically analyze the data. For categorical and continuous variables, appropriate descriptive statistical representations were created. The categorical variables (e.g., gender, religion, and awareness of the PMJAY and its components) were expressed as proportions and percentages with a 95% confidence interval. The continuous data (e.g., age) were checked using a Q-Q plot, and the mean (SD) was used for the normally distributed data, while the others were represented as the median (IQR). To know the association between the awareness of PMJAY and the level of education, gender, and occupation, the Pearson chi-square test was used, and the Fischer exact test was used whenever there was an expected cell count of any cell that was < 5. Overall statistical significance was attributed to P<0.05.

Ethical consideration

This study was approved by the Institute Ethics Committee, AIIMS, Patna (AIIMS/Pat/IEC/PGTh/Jan20/13). The principles of ethics were adhered to throughout the study and thereafter.

## Results

In our study, 268 (33.4%) of the participants were in the 60 years and above group, with a mean (SD) age of 49.5 (15.2) years. The majority of the study participants were male (725 [90%]). One-third of the study participants (289 [36%]) didn’t get any formal education. Most of the study participants were unskilled workers (288 [35.4%]). About 2/3rd of the 515 study participants (64.2%) belonged to the nuclear family. Nearly half of the study participants belonged to the lower middle class (338 [42.1%]). Almost all of the study participants belonged to the Hindu religion (785 [98%]). Around half of the study participants (441 [55%]) belonged to the general category. More than two-thirds of the 668 study participants (83.3%) were availing themselves of the PDS through ration cards (Table 1).

**Table 1:** Socio-demographic details (N=802) *Modified BG Prasad Classification (Feb 2021) [19] OBC: Other backward classes, SC: Schedule caste, ST: Schedule tribe

Variables	Categories	Frequency (n)	Percentage (%)
Age group (in years)	< 30 years	73	9.1
	30-45 years	240	29.9
	45-59 years	221	27.6
	≥ 60 years	268	33.4
Gender	Male	725	90
	Female	77	10
Education	No formal education	289	36.0
	Primary school	42	5.2
	Middle school	82	10.2
	Secondary school	243	30.3
	Senior secondary school	72	9.0
	Graduate and above	74	9.2
Occupation	Unemployed	139	17.1
	Unskilled worker	288	35.4
	Semiskilled worker	30	3.74
	Skilled worker	102	12.5
	Clerical, shop owner	203	25.0
	Semi-professional	13	1.6
	Professional	27	3.3
Type of family	Nuclear family	515	64.2
	Joint family	287	35.8
Socio-economic status^*^	Upper class	25	3.1
	Upper middle class	78	9.7
	Middle class	155	19.3
	Lower middle class	338	42.1
	Lower class	206	25.7
Religion	Hindu	785	98
	Muslims	17	2
Category	General	441	55.0
	OBC	139	17.3
	SC	199	24.8
	ST	23	2.9
Ration card	Present	668	83.3
	Not present	134	16.7

**Figure 1:**
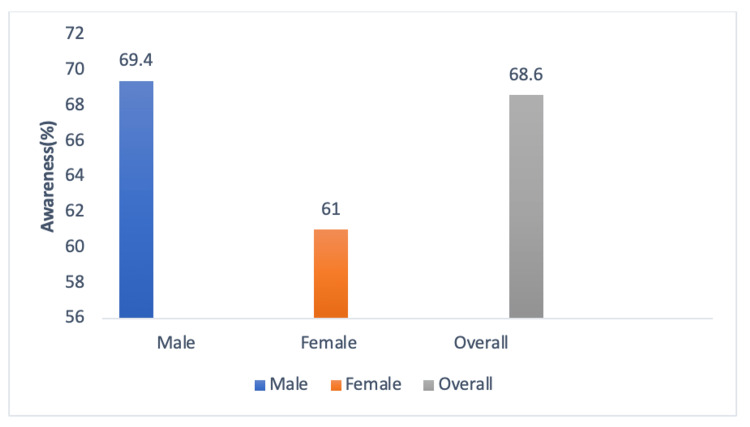
Level of awareness across genders compared with overall awareness

**Figure 2:**
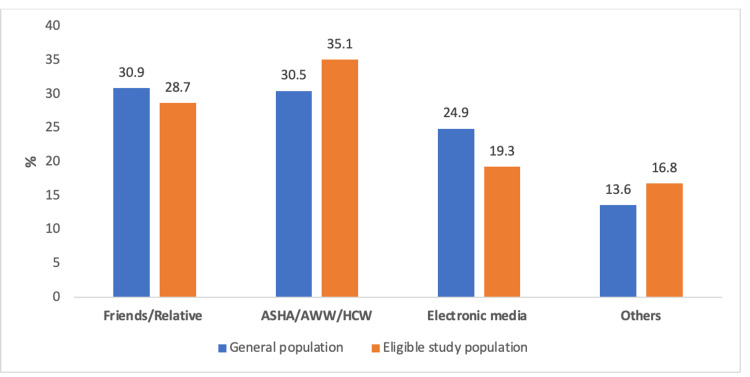
The primary source of information for the general population and eligible study population *ASHA=Accredited social health activist, AWW=Anganwadi worker, HCW=Health care worker. Others include the Internet, medical camps, and government pamphlets

Half of the study participants (49%) were not aware of health insurance, while awareness of the Ayushman Bharat scheme was 49.3% (45.8-52.71), but after proving individual components of the Ayushman Bharat scheme, awareness was found to be 68.6% (65.3-71.72) (Figure 1) while, out of 459 eligible study participants, 362 (78.9%) were aware of the Ayushman Bharat-PMJAY. The eligible study participants are assessed using six deprivation criteria and automatic inclusion (destitute, living on alms, manual scavenger households, primitive tribal groups, and legally released bonded labor) under the AB-PMJAY. The primary source of information in the general population was through friends/relatives (30.9%), while eligible study participants got information mostly through ASHA/AWW/HCW 35.1% (Figure 2).

The majority of study participants' primary source of information were friends/relatives 170 (30.9%), followed by ASHA/AWW/HCW that is 168 (30.5%), while in eligible study participants, the majority of eligible study participants' primary source of information was ASHA/AWW/HCW 127(35.1%). Of the 550 participants who were aware of the Ayushman Baharat-PMJAY among them, the majority of the participants were aware of the coverage amount 396 (72%), followed by card portability 173 (31.5%), a treatment package 72 (13.1%), and diagnostic covered (12.7%), while among the 362 eligible study participants who were aware of the Ayushman Bharat-PMJAY, the majority of the participants were aware of the coverage amount 275 (76%), followed by card portability 124 (34.3%), a treatment package 55 (15.2%), and diagnostic covered 53 (14.6%). The majority of the study participants, 474 (86.2%), were not aware of the eligibility criteria for Ayushman Bharat-PMJAY, while 37 (6.7%) knew that SC/ST households are eligible for the Ayushman Bharat health card. Of the eligible study participants, 303 (83.7%) were unaware of Ayushman Bharat-PMJAY, while 35 (9.7%) knew that SC/ST households are eligible for the Ayushman Bharat health card. Almost all of the participants were aware of the Ayushman Bharat health card; 524 (95.3%) and 354 (95.3%) of the eligible participants were aware of the Ayushman Bharat health card. The majority of the study participants 488 (88.7%), were aware that all members of the family are included in this Ayushman Bharat-PMJAY, whereas almost all eligible study participants 341 (94.2%) were aware that all members of the family are included in this Ayushman Bharat-PMJAY. Out of 524 participants who knew about the Ayushman Bharat card, 254 (46.2%) reported that the card can be availed from a government hospital, while 106 (13.2%) don’t know about its availability, and only 32 (5.8%) were aware of the charges of making the Ayushman Bharat card. Meanwhile in 354 eligible study participants who knew about the Ayushman Bharat card, 185 (51.1%) reported that the card can be availed from a government hospital, while 127 (35.1%) don’t know about its availability, while only 28 (7.7%) were aware of the charges of making the Ayushman Bharat card (Table 2).

**Table 2:** Awareness regarding the components of the Ayushman Bharat-PMJAY (N=802)

Variables	Categories	Aware study participants, n (%)	Aware and eligible study participant, n (%)
Components of Ayushman Bharat	Coverage Amount	396 (72)	275 (76)
	Card portability	173 (31.5)	124 (34.3)
	Treatment package	72 (13.1)	55 (15.2)
	Diagnostics covered	70 (12.7)	53 (14.6)
	Transportation expenses	29 (5.3)	21 (5.8)
	Knowledge of empaneled providers	27 (4.9)	20 (5.5)
	Post-discharge benefits	25 (4.5)	19 (5.2)
	Number of beneficiaries per family	17 (3.1)	13 (3.6)
	Addition of new family member.	11 (2)	4 (1.1)
	Grievance Mechanism	9 (1.6)	4 (1.1)
	Treatment without E-card	6 (1.1)	4 (1.1)
	Age limit of the dependents	4 (0.7)	1 (0.3)
Regarding the eligibility criteria of the families	Don't know about eligibility	474 (86.2)	303 (83.7)
	SC/ST households	37 (6.7)	35 (9.7)
	Landless families who derive major income from daily labor	29 (5.3)	16 (4.4)
	Others*	10 (1.2)	8 (2.2)
Ayushman Bharat health card	Ayushman Bharat health card	524 (95.3)	354(97.8)
Families covered in Ayushman Bharat-PMJAY	All members of the family	488 (88.7)	341 (94.2)
	Elderly	3 (0.5)	20 (5.5)
	Don't Know	59 (7.09)	1 (0.3)

Of 550 study participants, the majority, 444 (80.7%), reported that every family member should need a separate card, and the majority of study participants, 419 (76.3%), reported that it is necessary to own an Ayushman card for availing services. Most of the study participants, 297 (54%), reported that the Ayushman Bharat card can be used for emergency purposes. Only 173 (31.5%) of study participants were aware that cards could be used in states other than their own. In the pre-utilization part of the study majority of the participants, 394 (71.6%), were aware that there was no need for any premium payment, followed by 391 (71.1%), who were aware that there was no need for renewal of their card, and 389 (70.7%) were aware that pre-health check-ups are not needed in Ayushman Bharat health insurance. While of the 362 eligible study participants, the majority, 315 (87%), reported that every family member should need a separate card. In addition, approximately 292 people (80.7%) reported that an Ayushman card is required to access services. Most of the study participants, 173 (47.8%), reported that the Ayushman Bharat card can be used for emergency purposes. And only 117 (32.3%) of the study participants were aware that cards can be used in states other than the residential state. In the pre-utilization part, 283 (78.2%) were aware that there is no need for any premium payment, followed by 280 (77.3%) who were aware that there is no need for card renewal, and 274 (75.7%) were aware that pre-health check-ups are not needed in Ayushman Bharat health insurance (Table 3).

**Table 3:** Awareness regarding the pre-utilization process and the utilization of Ayushmann Bharat card facilities (n=550)

	Utility	Aware study participants, n (%)	Aware and eligible study participants, n (%)
Utilization of Ayushman Bharat-PMJAY	Need of separate card for each family member	444 (80.7)	315 (87)
	Need of Ayushman Bharat card always in hand to avail of benefits.	419 (76.3)	292 (80.7)
	Ayushman Bharat card is useful during emergency treatment.	297 (54.0)	215 (59.4)
	Usage of Ayushman Bharat Health Insurance Scheme in other than a residential state.	173 (31.5)	117 (32.3)
Pre-utilization of Ayushman Bharat-PMJAY	Renewal is not needed	394 (71.6)	283 (78.2%)
	No Premium money is needed for the AB-PMJAY	391 (71.1)	280 (77.3%)
	A pre-health check-up is not necessary to become eligible for Ayushman Bharat Health Insurance	389 (70.7)	274 (75.7)

Of the study participants who are eligible and aware of the majority of the families, 213 (58%) have the Ayushman Bharat card, but the utilization of the Ayushman Bharat scheme was found to be very low, as only five (1.3%) study participants have used the facility. Three of the five study participants used it for cataract surgery, one for ear surgery, and one for a fractured leg. All have availed themselves of their services from the private health facility, and all are located in the urban area of Patna. Of the total five participants, two had to incur out-of-pocket expenses of INR 15000 and INR 500, which they spent on travel expenses and extra medical expenses.

Awareness of the Ayushman Bharat scheme showed a significant association with the age group and category of the eligible study participants, ration card holders, and the occupation of the study participant (Table 4).

**Table 4:** Association of the awareness of the eligible study participants with various variables (n=459) a: For the application of statistical tests, education groups are regrouped into three categories. b: The Fischer exact test was used as the expected cell count was less than c: For the statistical test, the upper middle class and upper class regrouped to make the upper class, and the lower middle class and lower class regrouped to make the lower class. d: For statistical test application occupation groups are regrouped into four categories. #Modified BG Prasad Classification (Feb 2021) [19] OBC: Other backward classes, SC: Schedule caste, ST: Schedule tribe *p-value by chi-square

Variables	Categories	Awareness of the eligible study participants		p-value*
		Yes, n (%)	No, n (%)	
Age group	< 30 years	31 (64.6)	17(35.45)	0.039
	30-45 years	129(80.6)	31(19.4)	
	45-60 years	106(77.4)	31(22.6)	
	≥60 years	362(78.9)	97(21.1)	
Education level	No formal education	134(75.3)	44(24.7)	0.296
	Education up to 10th class	178(80.5)	43(19.5)	
	Education above 10th class	50(83.3)	10(16.7)	
Gender	Male	331(77.9)	94(22.1)	0.080
	Female	31(91.2)	3(8.8)	
Religion	Hindu	351(78.5)	96(21.5)	0.475
	Muslim	11(91.7)	1(8.3)	
Category	General	118(72.4)	45(27.6)	0.015^b^
	OBC	68(89.5)^x^	8(10.5)	
	SC	159(80.7)^y^	38(19.3)	
	ST	17(73.9)^z^	6(26.1)	
Type of family	Joint Family	48(81.4)	11(18.6)	0.093
	Nuclear Family	245(76.3)	76(23.7)	
	Three-Generation Family	69(87.3)	10(12.7)	
Socio-economic status^c#^	Upper class	23(76.7)	7(23.3)	0.850
	Middle class	53(76.8)	16(23.2)	
	Lower class	286(79.4)	74(20.6)	
Occupation^d^	Unemployed	58(95.1)	3(4.9)	<0.0001^b^
	Skilled and Unskilled workers	233(73.7)	83(26.3)	
	Farmers and shopkeepers	64(87.7)	9(12.3)	
	Semi-professional and above	7(77.8)	2(22.2)	
Ration card	Present	333(82.4)	71(17.6)	<0.0001
	Not present	29(52.7)	26(47.3)	

## Discussion

This study was a community-based cross-sectional study conducted in a rural population of a village in Bihar, Eastern India, among 802 study participants. Very few similar studies are done among the rural population of India [21,22]. In our study, only half (51%) of the participants were aware of health insurance. Similar studies in other parts of the country reported a quite higher level of awareness, ranging from 74%-82% [21,23,24]. Another study from Bihar reported a similar awareness of 45% [25]. A study from Bhaktapur reported as high as 87% awareness regarding health insurance among the population [26]. This may be attributable to the fact that we studied the rural population, whereas other studies are either done at hospitals or in urban areas where awareness is higher than in their counterparts. A clear geographical distribution can be observed, with the southern states being more aware of health insurance as compared to the northern states. This can be attributed to the change in the sociocultural aspect and the difference in the literacy rate among the states.

In the current study, almost 68% of study participants were aware of the AB-PMJAY, which is higher compared to the studies done in the other northern states of India [22,27]. A study from rural Jammu reported awareness of the AB-PMJAY scheme as low as 28%, while in contrast, a study from rural Tamil Nadu reported the awareness to be 77% [15,22]. The awareness of the national health insurance scheme in Ilorin, Nigeria, was 78.9% [28]. This difference in the level of awareness can be attributed to the difference in socio-demographic characteristics and cultural characteristics between the states, which are in different geographical locations within India. There is an improvement in the level of awareness regarding AB among the rural population compared to 2019 when awareness was only 9.9% [27].

In the present study, the primary source of information for the AB-PMJAY health insurance scheme was reported to be Friends/Relatives (30.9%), followed by front-line workers like ASHA/AWW/HCW (30.5%). The contribution from electronic media and other forms of advertisement was less. A similar finding was observed in a study by Shet et al. [29] and Yellaiah et al. [30]. A similar study done by Gosh et al. (2013) reported that 24% of study participants got information about health insurance through the media and 26% from their friends [31]. In the rural setting, people are more involved in farming and also take part in social interaction through gatherings in the village, like at the temple or chapel; hence, family/friends play an important role in the spreading of information. Also, ASHA does visit the villages regularly for various purposes, including the distribution of the Prime Minister's Letter of Ayushman Bharat, because of which they were the major primary source of information for the Ayushman Bharat-PMJAY.

In this study, 31% of the study participant were aware of card portability, 72% of the study participants were aware of the coverage amount, 54% of study participants were aware that this scheme can also be used in emergencies, and only 4.5% and 1.6% of the participants were aware of the post-discharge benefits and the grievance mechanism, respectively. A similar study done by Pugazhenti et al. (2021) in the Thanjavur district of Tamil Nadu reported that 42% of the study participants were aware of the coverage amount, 23% of study participants were aware of the card portability, and 29% and 15% of the study participants were aware of the post-discharge benefits and grievance mechanism of the Ayushman Bharat health scheme, respectively [16]. The level of awareness in the present study has come lower for all components except the coverage amount. This can be attributed to the presence of the state chief minister's comprehensive health insurance scheme (CMCHIS), which later got integrated into the Ayushman Bharat health scheme in Tamil Nadu, while in Bihar there was no previous state-specific system as in the public health system in Tamil Nadu and the high literacy rate in Tamil Nadu. The eligible study participants having the ration card showed statistical significance with the awareness of the Ayushman Bharat-PMJAY; this could be due to the need for the ration card for the making of the Ayushman Bharat-PMJAY card. There was a significant association between both, and awareness was highest among the unemployed study participants (95.1%) as compared to the other participants. Due to the COVID-19 pandemic, many people had lost their jobs, which can be the reason for a higher level of awareness among the unemployed study participants.

In the present study, utilization of the Ayushman Bharat Health Scheme has come to a very low level-only, five study participants (1.3%) have used it, and all have used it for surgical treatment. Despite having the Ayushman Bharat-PMJAY, two study participants had to spend extra money. A similar study conducted in the rural area of Chennai reported utilization of Ayushman Bharat-PMJAY 60 (47.2%), of which 10 (16.67%) were used for medical conditions and 31 (51.66%) for surgical conditions, while 19 (31.67%) for both medical and surgical conditions. Despite using the Ayushman Bharat scheme, six (10%) households had to spend extra money [15], which is a higher rate of utilization than the present study, as this could be due to the COVID-19 pandemic, which severely affected routine and non-COVID-19 medical services in Bihar state.

Strength and limitation

This study was conducted in a community setting in the rural part of Bihar, and the response rate was 100% despite the ongoing COVID-19 pandemic. The sample size of the study was 802, and the maximum were the decision makers of the household. But as the study was conducted in a single block of Bihar and hence cannot be representative of the whole of India, and awareness levels might have been impacted due to the ongoing COVID-19 pandemic, where frontline workers were giving priority to COVID-19 services, and utilization of Ayushman Bharat services couldn’t be assessed adequately as the process of making cards is still in progress.

## Conclusions

Friends and relatives were the primary source of information for two out of every three rural community members who were aware of the Ayushman Bharat scheme. Among the eligible study participants, nearly three-fourth were aware of the Ayushman Bharat scheme, and the major source of information was accredited social health activists (ASHA)/anganwadi workers (AWW)/healthcare workers (HCW). The participants’ awareness of the various components of the Ayushman Bharat-PMJAY was varied and low. There was a significant association between occupation, category, and ration card status of eligible participants with the awareness of Ayushman Bharat-PMJAY. Creating awareness should be improved through Ayushman Bharat-PMJAY advertisement using IEC material and telecommunication, as well as regular strengthening of the health care worker network at the grass-root level like ASHA and AWW to improve the connection in the community. Identification of the eligible participants should be carried out for better utilization of the Ayushman Bharat-PMJAY.
